# The Comparative Effects of Rhus Coriaria and Triamcinolone in Patients with Recurrent Aphthous Stomatitis: A Single-Blinded Randomized Controlled Clinical Trial

**DOI:** 10.1155/2022/5855067

**Published:** 2022-10-10

**Authors:** Fatemeh Lavaee, Marzieh Ghasemi, Mohammad Amin Amiri, Nima Farshidfar

**Affiliations:** ^1^Oral and Dental Disease Research Center, Oral and Maxillofacial Disease Department, School of Dentistry, Shiraz University of Medical Sciences, Shiraz, Iran; ^2^Student Research Committee, Shiraz University of Medical Sciences, Shiraz, Iran; ^3^Orthodontic Research Center, School of Dentistry, Shiraz University of Medical Sciences, Shiraz, Iran

## Abstract

**Background:**

Recurrent aphthous stomatitis (RAS) is a common oral lesion with unknown etiology. Several treatment strategies are introduced for the treatment of RAS. In this regard, the therapeutic effects of Rhus coriaria, as one of the potential treatments, have recently caught attention. Since the clinical efficacy of Rhus coriaria has not been examined adequately. This study aims at evaluating the therapeutic effects of Rhus coriaria among patients with RAS.

**Method:**

s. Twenty-two patients with RAS were divided into two groups (*n* = 11). The experimental group received three pills of Rhus coriaria daily for 6 days, while the control group received triamcinolone (oral paste) three times a day for 6 days. The pain and size of the lesion were measured on the 1^st^, 2^nd^, 3^rd^, 4^th^, 5^th^, and 6^th^ days. The data were analyzed by SPSS 16. In this regard, Student's *t*-test and Sidak pairwise tests were used for assessment of inter and intragroup comparisons of the pain and the size of the lesion, respectively.

**Results:**

Intergroup comparisons indicate that there is no difference between the experimental and the control group (*p* > 0.05). Whereas, the intragroup analysis of the pain revealed significant changes (*p* < 0.05) in most of the time points for both groups. Besides, the intragroup analysis of the lesion size, showed significant changes in all the time points in the experimental group (*p* < 0.05). The results in the control group exhibited the same pattern, except on 1-5, 1-6, 2-6, and 3-6 intervals in the control group.

**Conclusions:**

The application of Rhus coriaria could significantly reduce lesion size and pain in patients with RAS. Accordingly, Rhus coriaria can be an effective medication for RAS treatment.

## 1. Introduction

Recurrent aphthous stomatitis (RAS) is a very common and painful mouth ulcer which usually affects labial mucosa, buccal mucosa, and tongue [1]. Aphthous ulcers are well-demarcated, shallow, recurrent round, or oval ulcers on nonkeratinized mucosa in immune-sufficient people [1]. They have necrotic center with a yellow-gray pseudomembrane and slightly raised red margins. Moreover, there are three types of RAS (minor, major, and herpetic forms) that can be clinically identified [1]. The main causes of RAS are unknown; however, some triggers for RAS outbreaks are introduced such as emotional stress, deprivation of sleep, trauma, lack of different types of vitamin B, iron and folic acid deficiencies, menstruation, anemia, and fluctuation of sex hormones in women [2, 3]. Nevertheless, the most common treatment for RAS is symptom therapy which is focused on reducing pain [4].

Recently, global attention has been paid to the introduction of new herbal medications for RAS treatment. Rhus coriaria, commonly called Sicilian sumac, is a plant from the anacardiaceous family with a height of three to five meters, white branches, and fluffy circular fruit. With its various geographical climates, Iran presents appropriate conditions for the growth of Rhus coriaria [5]. This plant has been used in herbal medicine due to its various properties such as antimicrobial, antioxidant, hypoglycemic, hypolipidemic, antimutagenic, antimigratory, and anti-ischemic activities [6].

Due to the aforementioned properties of Rhus coriaria, the effect of its extract has already been investigated in many different studies on oral environment [2–5]. On this basis, in a study by Kermanshah et al. [7], the hydroalcoholic extracts of some plants including the fruit of Rhus coriaria were demonstrated to have antibacterial activity against *Streptococcus mutans*, *Lactobacillus rhamnosus, and Actinomyces viscosus* which play an important role in dental and periodontal diseases [8, 9]. In addition, in a study by Vahid-Dastjerdi et al. [10], the antiplaque effects of Rhus coriaria fruit water extracts were assessed against plaque formation on the orthodontic tooth wires. They [10] demonstrated the inhibitory effect of Rhus coriaria on dental plaque accumulation on the orthodontic wire. In addition, Rhus coriaria exhibited a significant antibacterial effect against *Streptococcus mutants*. In addition, the analgesic and healing effects of Rhus coriaria are also investigated in several studies [11, 12]. In a study by Mohammadi et al. [11], the analgesic effects of hydroalcoholic Rhus coriaria leaf extract were investigated in an animal model. The results showed antinociceptive activity at 300 mg/kg hydroalcoholic Rhus coriaria [11]. Choosing a specific herbal composition should be accompanied by knowing its accurate ingredients and medical effect which can be helpful in pain relief and wound healing.

The analgesic effect of Rhus coriaria potentiates its application for several inflammatory lesions, especially in the oral mucosa. Based on the results of the mentioned studies, the authors of this study hypothesized that utilization of Rhus coriaria can be effective in the treatment of RAS. Since the clinical efficacy of Rhus coriaria has not been examined adequately, this study aims at evaluating the therapeutic effects of Rhus coriaria among patients with RAS.

## 2. Materials and Methods

### 2.1. Trial Design

The study was designed as a two-arm and single-blind randomized controlled clinical trial, applying a parallel design with a 1 : 1 allocation ratio. There were no changes in methods after trial commencement. It is worth mentioning that this study was conducted according to the CONSORT (Consolidated Standards of Reporting Trials) 2010 guideline.

### 2.2. Sample Size

The sample size was determined by a statistician considering a confidence interval of 95% and power of 80%. The required sample size was calculated to be 11 participants per group.

### 2.3. Participants

87 patients attending the oral medicine clinic of the Shiraz University of Medical Sciences, from January 2017 to December 2018, with a diagnosis of minor RAS were evaluated for the eligibility criteria. The patients with RAS from the first day of its appearance were enrolled in this research. The patients with minor RAS in the first day of recurrence were included in this study. Other inclusion criteria are not using any analgesic mouthwash, oral pastes, or systemic analgesia for 3 days before this evaluation and any topical or oral corticosteroid or colchicine since a month before, no history of related systemic diseases such as Crohn's disease, Behcet's disease, Reiter syndrome, or other inflammatory disease and allergic reaction to Rhus coriaria.

### 2.4. Drug Preparation

Rhus coriaria L. fruits were purchased from the local market in Shiraz city. Plant material was identified by a botanist at the Department of Phytopharmaceuticals, School of Pharmacy, Shiraz University of Medical Sciences. A voucher specimen was deposited in the Shiraz School of Pharmacy collection (Registered Number: PM 533). Plant material was air-dried under shade for 28 days (Temperature 20–37°C) before being powdered in a hammer mill and sieved through 250 mm mesh. The powder was then used to prepare 9 mm pills. The composition of the pills was 50% Avicel as an ideal granulation binder, 0.2% magnesium stearate, and 49.8% Rhus coriaria powder.

### 2.5. Randomization, Blinding, and Allocation Concealment

Twenty-two eligible patients, who signed a written consent form, were randomly allocated to two parallel groups by the clinic secretary, who had been instructed on applying a statistically randomized list. The randomized list was generated using the block randomization method. Neither the clinicians nor the researchers were blinded to the allocation of the patients. Based on the different types of drugs, the patients were not blinded to the drug allocation as well. However, the statistician was the only person who was blinded to the allocation of the patients since he just received the data with the label of A and B groups without the disclosure of treatment groups.

### 2.6. Interventions

In the experimental group, patients were instructed to take three pills of Rhus coriaria daily for 6 days. In this regard, patients were instructed to apply the pill on the lesions and hold the pills for several minutes until they were solved completely. On the other hand, in the control group, the other patients were instructed to administer the oral paste form of triamcinolone 0.1% (Triadent, Raha Pharma Corporation, Isfahan, Iran) three times a day for 6 days. All patients were also recommended to make no change in their previous state of physical activity and diet for the course of study. They were also advised to report any side effects to the on-call physician via telephone conversation.

### 2.7. Outcomes

The clinicians and researchers evaluated the visual analog scale (VAS) of the patients before and after using the pills of Rhus coriaria and oral paste of triamcinolone on the 1^st^, 2^nd^, 3^rd^, 4^th^, 5^th^, and 6^th^ days. The size of the lesions in each patient was also measured on the 1^st^, 2^nd^, 3^rd^, 4^th^, 5^th^, and 6^th^ days by a graded tongue blade.

### 2.8. Statistical Analysis

The descriptive data are presented as means and standard deviations. In this regard, Student's *t*-test and Sidak pairwise tests were used for assessment of inter and intragroup comparisons of the pain and the size of the lesion, respectively. A *p*-value of less than 0.05 was deemed significant. The data were analyzed with IBM SPSS 22.

### 2.9. Ethical Consideration

The study protocol was in compliance with the Declaration of Helsinki and approved by the Ethics Committee of Shiraz University of Medical Sciences (Reference number: IR.SUMS.REC.1396.S160).

## 3. Results

### 3.1. Baseline Characteristics and Study Flow

**Figure 1:**
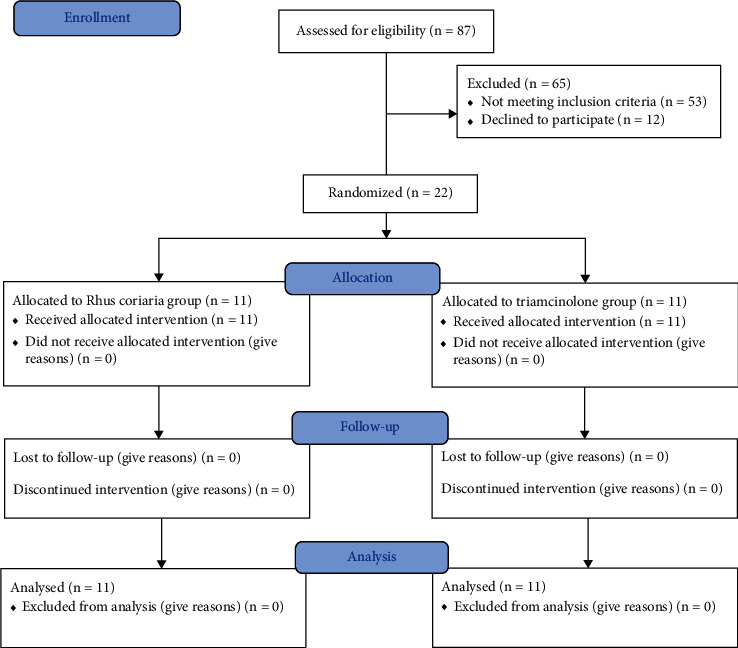
CONSORT Flow diagram of the study inclusion, allocation, and follow up.

From January 2017 to December 2018, a total of 87 patients were assessed for eligibility and, finally, 22 of them were randomized to receive either the trial drug or conventional one (11 patients in each group). No patients discontinued their treatments during the study; therefore, all the 22 patients were included in the final analysis.  Figure 1 shows a flowchart of patient enrolment, randomization, and outcomes. Additionally, demographic information of patients with RAS in both experimental and control groups is illustrated in Table 1.

### 3.2. Outcome Measures

The changes of the values are the following: (1) VAS change, and (2) Size of the lesion were compared between the experimental and control groups in each day (intergroup comparison); consequently, there were no significant differences between the experimental and control groups in terms of those values (Table 2).

Aside from the intergroup comparison, intragroup comparison was also done for evaluating the differences in either of the experimental and control groups for all time periods (Tables 3 and 4). In this regard, the results showed significant differences in either of the experimental and control groups in terms of the aforementioned values changes (VAS change and Size of the lesion) for almost all time periods. However, the exceptions are shown with a superscript star (^∗^) in each row of the Tables 3 and 4.

### 3.3. Safety Measures

No harms or unintended effects in any of patients in either experimental or control groups were observed during the study.

## 4. Discussion

Nowadays, corticosteroids, including triamcinolone acetonide are considered as the preferred and conventional [6] treatment option for lichen planus [7–9] since they can relieve the signs and symptoms through lowering the inflammation of the lesion [10, 11]. Moreover, it was found that 0.1% triamcinolone acetonide orabase can repair the imbalance of oxidation/antioxidation condition in the oral environment which is considered as a contributing factor in developing the oral lichen planus condition [12, 13]. Worth mentioning, according to the current studies [14–16], the motivation of developing an alternative treatment option other than corticosteroids is the due to their several adverse effects, such as telangiectasia, insomnia, fatigue, fluid retention, nausea, suppressed immune activity, mood swings, dry mouth, oral mucosa thinning, and candidiasis in the oral cavity [12, 17]. Owing to the downsides of these medications attempts have been made to find out an alternative treatment with less side effects and higher effectiveness which can provide the patient with higher quality of life.

According to the results of this study, there was no statistically significant difference between the oral paste of triamcinolone and Rhus coriaria in terms of the changes in the size of the lesion and the VAS scores. In the intragroup analysis, it was shown that the VAS change between the first day and second day was statistically significant in the group treated with Rhus coriaria which indicates the considerable impact of Rhus coriaria on the first day of intervention. Concerning the intragroup comparison of lesion size changes between the groups, it was demonstrated that the group treated with Rhus coriaria exerted significant changes between all the time points. However, the same result was not seen in the control group. The intragroup comparisons indicate the effectiveness of both Rhus coriaria and triamcinolone acetonide on RAS during the 6-day treatment period; however, Rhus coriaria showed more significant changes and faster VAS decrease. Due to these encouraging findings, further studies are required to validate these results.

According to the literature [10, 13, 14], there are several factors that can explain the effectiveness of Rhus coriaria on a lesion, such as RAS which has an autoimmune nature [15]. Studies have shown the bacterial population in patients with RAS is different from the normal population [16–18]. The current finding suggests the potential correlation of the unusual bacterial population with the disease pathology [16–18]. Worth mentioning, one of the effects of Rhus coriaria is its antibacterial impact which has been tested on the pathogenic bacteria of oral cavity [5, 10, 14]. This phenomenon can partly explain the possible effect of Rhus coriaria on RAS.

Another noticeable impact of Rhus coriaria is its anti-inflammatory and neuroprotective effect [13]. In this regard, Khalilpour et al. [13] have suggested scavenging free radicals as a mechanism of Rhus coriaria's anti-inflammatory effect. Also, in order to discover the chemical components responsible for Rhus coriaria's antioxidant activities, they [13] undertook a phytochemical screening. According to the results [13], phenolics and flavonoids are the chemical agents responsible for this phenomenon since these molecules can deactivate the oxidants and prevent the pathological conditions, such as autoimmune disorders and so forth. Of all the chemical components in Rhus coriaria extract, Linoleic acid is found to be the most prevalent fatty acid [13]. Since the 400 mg/kg linoleic acid was found to be more effective than the extract of Rhus coriaria in preventing ischemia in a rat model, it was suggested as the major component of Rhus coriaria responsible for its anti-inflammatory effects [13].

On the other hand, several studies [15, 19] have discussed the autoimmune nature of RAS. Several factors are known to modify the immunologic response during aphthous pathogenesis. Aside from the cascade of proinflammatory cytokines observed in this regard, the considerable leukocytes infiltration is the characteristic of this lesion [15]. Therefore, one of the main aspects of the medications that can alleviate the signs and symptoms of this lesion should be their anti-inflammatory impact to dwindle the severity of the lesion. This is why both the triamcinolone as a corticosteroid and Rhus coriaria's extract were both effective in this regard [13, 20].

According to the biochemical analysis of the components of Rhus coriaria [13], it can be concluded that this substance exerts its impact on aphthous lesions by exerting both the anti-inflammatory and antibacterial effects. Although firm conclusions cannot be drawn from our single study, our intervention has shown promising outcomes concerning the Rhus coriaria's effect on oral lesions. Therefore, by discussing the possible molecular basis of this clinical effect, we strongly suggest further large-scale studies to confirm our results. In addition, since several complications are addressed with the use of corticosteroids, such as impeding the hormonal balance, suppression of immune responses, enhancing the risk of infections, and so on [20], finding a highly efficient alternative can have a much more beneficial effect on the patients' quality of life.

## 5. Conclusion

Within the limitations of this study, following conclusions can be drawn:

- Rhus coriaria can be a highly efficient treatment for RAS
- the effect of Rhus coriaria on RAS wasn't different from the triamcinolone oral paste in all the time points
- the intragroup analysis of the size of the lesion showed that Rhus coriaria could result in significant differences between all time-points while the same result wasn't established for the control group
- the intragroup analysis of the VAS scores showed significant differences between most of the time-points in both groups

## Figures and Tables

**Table 1:** Demographic information of patients with RAS in both experimental and control groups.

Characteristics	Experimental group	Control group
Gender (F/M)	7/4	6/5
Age	34.5 ± 2.1	33.7 ± 1.5

**Table 2:** Intercomparison of the values of VAS change and the size of the lesion on days 1 to 6 between the experimental and control groups.

VAS change (day)	Group	*N*	Mean	Std. deviation	*p* value	Size of the lesion (day)	Group	*N*	Mean	Std. deviation	*p* value
VAS Change (1)	Experimental	11	-2.6667	1.58274	0.327	Size of the Lesio*n* (1)	Experimental	11	2.0385	1.04526	0.549
	Control	11	-2.1282	1.07616			Control	11	1.9727	0.62304	

VAS Change (2)	Experimental	11	-1.6944	0.79720	0.146	Size of the Lesio*n* (2)	Experimental	11	1.7577	1.08970	0.965
	Control	11	-2.0769	0.75955			Control	11	1.7409	0.68294	

VAS Change (3)	Experimental	11	-1.6667	1.02494	0.236	Size of the Lesio*n* (3)	Experimental	11	1.4346	1.15675	0.600
	Control	11	-1.9231	0.49355			Control	11	1.3375	0.56734	

VAS Change (4)	Experimental	11	-1.4167	1.08362	0.389	Size of the Lesio*n* (4)	Experimental	11	1.0000	1.00830	0.869
	Control	11	-1.1538	0.80064			Control	11	1.0833	0.46482	

VAS Change (5)	Experimental	11	-0.6667	0.77850	0.794	Size of the Lesio*n* (5)	Experimental	11	0.6154	0.79461	0.810
	Control	11	-0.6154	0.96077			Control	11	0.6917	0.50535	

VAS Change (6)	Experimental	11	-0.2500	0.45227	0.573	Size of the Lesio*n* (6)	Experimental	11	0.4231	0.82981	0.775
	Control	11	-0.1538	0.55470			Control	11	0.3708	0.39339	

**Table 3:** Intracomparison of VAS changes between each two days in both experimental and control groups.

Group	VAS change (day)		Mean	Std. deviation	Sig. (2-tailed)
Experimental	Pair 1	Change (1)	-2.6667	1.58274	0.037
		Change (2)	-1.6944	0.79720	
	Pair 2	Change (1)	-2.6667	1.58274	0.124^∗^
		Change (3)	-1.6667	1.02494	
	Pair 3	Change (1)	-2.6667	1.58274	0.095^∗^
		Change (4)	-1.4167	1.08362	
	Pair 4	Change (1)	-2.6667	1.58274	0.005
		Change (5)	-0.6667	0.77850	
	Pair 5	Change (1)	-2.6667	1.58274	<0.001
		Change (6)	-0.2500	0.45227	
	Pair 6	Change (2)	-1.6944	0.79720	0.934^∗^
		Change (3)	-1.6667	1.02494	
	Pair 7	Change (2)	-1.6944	0.79720	0.444^∗^
		Change (4)	-1.4167	1.08362	
	Pair 8	Change (2)	-1.6944	0.79720	0.003
		Change (5)	-0.6667	0.77850	
	Pair 9	Change (2)	-1.6944	0.79720	<0.001
		Change (6)	-0.2500	0.45227	
	Pair 10	Change (3)	-1.6667	1.02494	0.053^∗^
		Change (4)	-1.4167	1.08362	
	Pair 11	Change (3)	-1.6667	1.02494	0.001
		Change (5)	-0.6667	0.77850	
	Pair 12	Change (3)	-1.6667	1.02494	<0.001
		Change (6)	-0.2500	0.45227	
	Pair 13	Change (4)	-1.4167	1.08362	0.001
		Change (5)	-0.6667	0.77850	
	Pair 14	Change (4)	-1.4167	1.08362	<0.001
		Change (6)	-0.2500	0.45227	
	Pair 15	Change (5)	-0.6667	0.77850	0.019
		Change (6)	-0.2500	0.45227	

Control	Pair 1	Change (1)	-2.1282	1.07616	0.835^∗^
		Change (2)	-2.0769	0.75955	
	Pair 2	Change (1)	-2.1282	1.07616	0.582^∗^
		Change (3)	-1.9231	0.49355	
	Pair 3	Change (1)	-2.1282	1.07616	0.047
		Change (4)	-1.1538	0.80064	
	Pair 4	Change (1)	-2.1282	1.07616	0.016
		Change (5)	-0.6154	0.96077	
	Pair 5	Change (1)	-2.1282	1.07616	<0.001
		Change (6)	-0.1538	0.55470	
	Pair 6	Change (2)	-2.0769	0.75955	0.827^∗^
		Change (3)	-1.9231	0.49355	
	Pair 7	Change (2)	-2.0769	0.75955	0.038
		Change (4)	-1.1538	0.80064	
	Pair 8	Change (2)	-2.0769	0.75955	0.001
		Change (5)	-0.6154	0.96077	
	Pair 9	Change (2)	-2.0769	0.75955	<0.001
		Change (6)	-0.1538	0.55470	
	Pair 10	Change (3)	-1.9231	0.49355	0.001
		Change (4)	-1.1538	0.80064	
	Pair 11	Change (3)	-1.9231	0.49355	<0.001
		Change (5)	-0.6154	0.96077	
	Pair 12	Change (3)	-1.9231	0.49355	<0.001
		Change (6)	-0.1538	0.55470	
	Pair 13	Change (4)	-1.1538	0.80064	0.003
		Change (5)	-0.6154	0.96077	
	Pair 14	Change (4)	-1.1538	0.80064	<0.001
		Change (6)	-0.1538	0.55470	
	Pair 15	Change (5)	-0.6154	0.96077	0.008
		Change (6)	-0.1538	0.55470	

**Table 4:** Intragroup comparison of the size of the lesion between each two days in both experimental and control groups.

Group	Size of the lesion (day)		Mean	Std. deviation	Sig. (2-tailed)
Experimental	Pair 1	Size (1)	2.0385	1.04526	<0.001
		Size (2)	1.7577	1.08970	
	Pair 2	Size (1)	2.0385	1.04526	<0.001
		Size (3)	1.4346	1.15675	
	Pair 3	Size (1)	2.0385	1.04526	0.001
		Size (4)	1.0000	1.00830	
	Pair 4	Size (1)	2.0385	1.04526	0.002
		Size (5)	0.6154	0.79461	
	Pair 5	Size (1)	2.0385	1.04526	<0.001
		Size (6)	0.4231	0.82981	
	Pair 6	Size (2)	1.7577	1.08970	<0.001
		Size (3)	1.4346	1.15675	
	Pair 7	Size (2)	1.7577	1.08970	<0.001
		Size (4)	1.0000	1.00830	
	Pair 8	Size (2)	1.7577	1.08970	<0.001
		Size (5)	0.6154	0.79461	
	Pair 9	Size (2)	1.7577	1.08970	<0.001
		Size (6)	0.4231	0.82981	
	Pair 10	Size (3)	1.4346	1.15675	<0.001
		Size (4)	1.0000	1.00830	
	Pair 11	Size (3)	1.4346	1.15675	<0.001
		Size (5)	0.6154	0.79461	
	Pair 12	Size (3)	1.4346	1.15675	<0.001
		Size (6)	0.4231	0.82981	
	Pair 13	Size (4)	1.0000	1.00830	<0.001
		Size (5)	0.6154	0.79461	
	Pair 14	Size (4)	1.0000	1.00830	<0.001
		Size (6)	0.4231	0.82981	
	Pair 15	Size (5)	0.6154	0.79461	0.001
		Size (6)	0.4231	0.82981	

Control	Pair 1	Size (1)	1.9727	0.62304	<0.001
		Size (2)	1.7409	0.68294	
	Pair 2	Size (1)	1.9727	0.62304	0.004
		Size (3)	1.3375	0.56734	
	Pair 3	Size (1)	1.9727	0.62304	0.015
		Size (4)	1.0833	0.46482	
	Pair 4	Size (1)	1.9727	0.62304	0.061^∗^
		Size (5)	0.6917	0.50535	
	Pair 5	Size (1)	1.9727	0.62304	0.630^∗^
		Size (6)	0.3708	0.39339	
	Pair 6	Size (2)	1.7409	0.68294	<0.001
		Size (3)	1.3375	0.56734	
	Pair 7	Size (2)	1.7409	0.68294	<0.001
		Size (4)	1.0833	0.46482	
	Pair 8	Size (2)	1.7409	0.68294	0.032
		Size (5)	0.6917	0.50535	
	Pair 9	Size (2)	1.7409	0.68294	0.600^∗^
		Size (6)	0.3708	0.39339	
	Pair 10	Size (3)	1.3375	0.56734	<0.001
		Size (4)	1.0833	0.46482	
	Pair 11	Size (3)	1.3375	0.56734	<0.001
		Size (5)	0.6917	0.50535	
	Pair 12	Size (3)	1.3375	0.56734	0.057^∗^
		Size (6)	0.3708	0.39339	
	Pair 13	Size (4)	1.0833	0.46482	<0.001
		Size (5)	0.6917	0.50535	
	Pair 14	Size (4)	1.0833	0.46482	0.019
		Size (6)	0.3708	0.39339	
	Pair 15	Size (5)	0.6917	0.50535	<0.001
		Size (6)	0.3708	0.39339	

## Data Availability

The datasets of this study are available by the corresponding author on a reasonable request.
